# The Role of Natural Killer T Cells in Cancer—A Phenotypical and Functional Approach

**DOI:** 10.3389/fimmu.2018.00367

**Published:** 2018-02-27

**Authors:** Daniëlle Krijgsman, Marianne Hokland, Peter J. K. Kuppen

**Affiliations:** ^1^Department of Surgery, Leiden University Medical Center, Leiden, Netherlands; ^2^Department of Biomedicine, Aarhus University, Aarhus, Denmark

**Keywords:** natural killer T cells, cancer, tumor immunology, immune escape, immunotherapy

## Abstract

Natural killer T (NKT) cells are a subset of CD1d-restricted T cells at the interface between the innate and adaptive immune system. NKT cells can be subdivided into functional subsets that respond rapidly to a wide variety of glycolipids and stress-related proteins using T- or natural killer (NK) cell-like effector mechanisms. Because of their major modulating effects on immune responses *via* secretion of cytokines, NKT cells are also considered important players in tumor immunosurveillance. During early tumor development, T helper (T_H_)1-like NKT cell subsets have the potential to rapidly stimulate tumor-specific T cells and effector NK cells that can eliminate tumor cells. In case of tumor progression, NKT cells may become overstimulated and anergic leading to deletion of a part of the NKT cell population in patients *via* activation-induced cell death. In addition, the remaining NKT cells become hyporesponsive, or switch to immunosuppressive T_H_2-/T regulatory-like NKT cell subsets, thereby facilitating tumor progression and immune escape. In this review, we discuss this important role of NKT cells in tumor development and we conclude that there should be three important focuses of future research in cancer patients in relation with NKT cells: (1) expansion of the NKT cell population, (2) prevention and breaking of NKT cell anergy, and (3) skewing of NKT cells toward T_H_1-like subsets with antitumor activity.

## Key Points

- NKT cells comprise a unique subset of CD1d-restricted T cells with characteristics of both NK- and T cells that can be subdivided into functional subsets.
- NKT cells are able to switch between different functional subsets upon cell–cell interaction or interaction with signaling molecules.
- Activated NKT cells have a major regulatory effect on other immune cells *via* cytokine production and cell–cell interaction, which results in amplification or dampening of the immune response.
- T_H_1-like NKT cells have the potential to induce an antitumor response while T_H_2- and T_reg_-like NKT cell subsets facilitate immune escape and tumor progression.
- Overstimulation of NKT cells during tumor progression might lead to induction of anergy and skewing of NKT cells toward T_H_2-/T_reg_-like subsets, thereby facilitating tumor progression and immune escape.
- In cancer patients, there should be three important focuses of future research: (1) expansion of the NKT cell population, (2) prevention and breaking of NKT cell anergy, and (3) skewing of NKT cells toward T_H_1-like subsets with antitumor activity.

## Introduction

The immune system is a host defense mechanism that plays a pivotal role in the protection against pathogens and cancer (1). It comprises multiple specialized subsets of cells that differentiate from a common pluripotent progenitor, the hematopoietic stem cell (2). These subsets include natural killer T (NKT) cells that feature characteristics of both conventional T cells and natural killer (NK) cells. Upon activation, NKT cells are able to kill target cells either directly (3–5) or indirectly by influencing both myeloid- and lymphoid-derived immune cells (6). Moreover, NKT cells are potent immune regulators since they can skew immune responses toward both inflammation and tolerance very quickly by secreting either T helper (T_H_)1-, T_H_2-, T_H_17-, T regulatory (T_reg_)-, or follicular helper (T_FH_)-cell-associated cytokines (7). Because of their major modulating effects on immune responses, NKT cells have also been considered important mediators of tumor immunosurveillance (8). The role of NKT cells in relation to cancer has therefore been the focus of recent studies. In this review, we discuss the role of NKT cells in cancer in relation to their phenotype and function. We focus on non-hematological malignancies, i.e., carcinomas, sarcomas, melanomas, and neuroblastomas. First, the development and function of NKT cells are addressed in healthy individuals. Thereafter, the role of NKT cells is discussed in the development and progression of cancer. Finally, available NKT cell-based immunotherapies are presented and possibilities for future research are discussed.

## Development and Localization of NKT Cells

NKT cells constitute a unique, but highly heterogeneous, subset of immune cells that arise in the thymus from CD4^+^CD8^+^ cortical thymocytes that have undergone T cell receptor (TCR) gene rearrangement, as is the case with conventional T cells (9). TCRs are composed of an α- and a β-chain, each containing a variable and constant domain. The TCR α-chain is generated by recombination of the variable (V) and joining (J) segments, whereas the β-chain also requires diversity (D)-segment recombination. Based on their TCR repertoire, two NKT cell subsets have been described: type I and type II NKT cells. Type I NKT cells were first identified in mice in 1990 as a unique T cell population expressing the Vα14Jα18 invariant TCR α-chain. The type I NKT cell subset recognizes the glycosphingolipid α-galactosylceramide (α-GalCer) or its synthetic analogs when presented by major histocompatibility complex (MHC) class I-like CD1d molecules (10–12). Four years after the identification of the invariant Vα14Jα18 TCR α-chain in mice, the human counterpart Vα24Jα18 was discovered which predominantly pairs with the Vβ11 TCR β-chain (13–16). In addition to type I NKT cells, type II NKT cells are described with a more diverse and less well-defined TCR repertoire recognizing non-α-GalCer molecules (primarily sulfatide) presented by CD1d molecules (12, 17–19).

### Development of Type I NKT Cells in Mice

The development of type I NKT cells has been thoroughly studied in mice. During positive selection in the murine thymus, T cells expressing TCRs that are capable of binding to MHC class I or II molecules on cortical thymic epithelial cells are selected to undergo lineage commitment (9). This process leads to maturation of CD4^+^ or CD8^+^ T cells that recognize MHC-presented peptides. Alternatively, type I NKT cells that express the randomly rearranged invariant Vα14Jα18 chain are positively selected upon binding to CD1d molecules expressed by cortical thymocytes (9, 20–22). As a result of this alternative positive selection, they recognize lipid-derived antigens presented by CD1d molecules (10). During the maturation process, a part of the type I NKT cell population retains expression of the T cell-associated marker CD4, resulting in two major populations in mice: CD4^+^CD8^−^ and CD4^−^CD8^−^ (double negative, DN) type I NKT cells (23, 24). In addition, Type I NKT cells acquire expression of the natural killer receptor (NKR) NK1.1 during maturation (9).

### Development of Type I NKT Cells in Humans

Although the thymic development of type I NKT cells is well defined in mice, it has not as yet been studied in details in humans. It has been reported that NKT precursor cells can be identified in thymic tissue derived from human embryos and young children (25, 26). Similar to type I NKT cell development in mice, human type I NKT cells express cell surface markers that are usually associated with both T- and NK cells. For instance, a part of the human type I NKT cell population retains expression of the T cell-associated markers CD4 or CD8 during maturation, resulting in three major populations in humans: CD4^+^CD8^−^, CD4^−^CD8^+^, and DN type I NKT cells (27, 28). In addition, a part of the human type I NKT cells acquires expression of the NK cell-associated marker CD161 (the human counterpart of NK1.1 in mice), the classical NK cell marker in humans CD56 (27–29), and various other NK cell-associated receptors (27–31).

### Localization of Type I NKT Cells in Humans

After development and maturation in the thymus, NKT cells migrate to the periphery. In general, human type I NKT cells are present in small numbers (<0.1% of total T lymphocytes) in peripheral blood (PB), lymph nodes, spleen, thymus, lung, and bone marrow (32–34), whereas larger type I NKT cell populations reside in the liver, colon, kidney (~1% of total T lymphocytes) (35–37), and omentum (~10% of total T lymphocytes) (38). Importantly, it has to be taken into account that NKT cell numbers vary substantially among healthy individuals. For instance, circulating type I NKT cells have been reported to comprise more than 5% of the total T lymphocyte population in some individuals (39).

### Morphology of NKT Cells

Despite the fact that NKT cells are derived from the T cell lineage, their morphology resembles NK cells more closely. NK and NKT cells are both referred to as large granular lymphocytes, whereas T cells are described as small and non-granular (40–42). In addition, NKT cells were reported to have a low nuclear-to-cytoplasmic ratio and their nucleus contained dispersed chromatin, similar to NK cells (41, 43–45).

## Characterization and Identification of Human NKT Cell Subsets

In addition to classification of NKT cells in type I and type II NKT cells based on their TCR repertoire, human NKT cells can also be classified into functional subsets based on their cytokine secretion pattern upon activation, using a similar approach as for T_H_-cell subsets (7).

### Functional Type I NKT Cell Subsets

**Figure 1:**
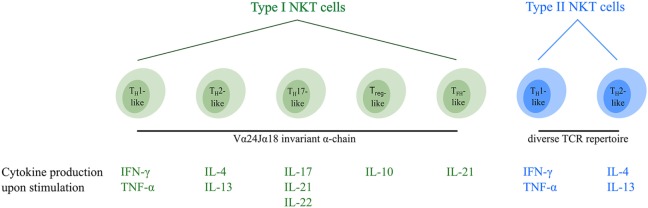
Overview of the different functional human NKT cell subsets. CD1d-restricted human NKT cells can be divided into subsets based on their TCR repertoire and cytokine profile. Type I NKT cells express the invariant Vα24Jα18 TCR α-chain and can be subdivided into five distinct functional subsets (indicated in green). In addition, type II NKT cells express a diverse TCR repertoire and can be subdivided into two functional subsets (indicated in blue). Upon activation, NKT cells secrete a unique pattern of cytokines, indicated for each subtype. Type I and type II NKT cells are able to switch between different functional subsets upon interactions within the TME. Abbreviations: NKT, natural killer T; TCR, T cell receptor; T_H_, helper T; T_reg_, regulatory T; T_FH_, follicular helper T; TME, tumor microenvironment.

At the moment, type I NKT cells can be divided into five different functional subsets (Figure 1). T_H_1-like type I NKT cells have been identified in healthy individuals producing T_H_1-associated cytokines such as IFN-γ and TNF-α upon stimulation (7, 30, 46). The majority of these type I NKT cells are DN and are thought to exert limited cytotoxic function (30, 46). They are able to induce an effective pro-inflammatory immune cascade through cytokine signaling. Furthermore, a second T_H_2-like type I NKT cell subset with regulatory properties has been described secreting IL-4 and IL-13 upon activation (7, 30, 46). This type I NKT cell subset mainly consists of CD4^+^CD8^−^ cells which are able to suppress immune responses in various disease models (47–49). Recently, three additional minor type I NKT cell subsets were identified. T_H_17-like type I NKT cells have been described, secreting the pro-inflammatory cytokines IL-17, IL-21, and IL-22 when activated (50, 51). In addition, FOXP3 expressing T_reg_-like type I NKT cells secreting the immunosuppressive cytokine IL-10 have been identified (52), as well as T_FH_-like type I NKT cells secreting IL-21 upon activation (46, 53). Interestingly, murine studies showed that functional type I NKT cell subsets (Figure 1) express unique transcription factors and the “choice” to become a certain subset appears to be set in the thymus during fetal development (54). The fate of type I NKT cells might, however, not be permanently determined at this time since their cytokine production upon activation can be influenced by the microenvironment (27, 55), similar to T_H_-cell subsets (56). For instance, the cytokine secretion pattern of type I NKT cells is altered by the presence of immunosuppressive cytokines and/or immune cell subsets in the tumor microenvironment (TME) (57), as well as costimulation *via* CD28 (58), thereby implying plasticity of type I NKT cell subsets.

### Identification of Type I NKT Cell Subsets

NKT cells can be identified from human peripheral blood mononuclear cells (PBMCs) with flow cytometry using monoclonal antibodies (mAb) and multimers as thoroughly described by Metelitsa (59). For instance, type I NKT cells have often been identified by costaining with anti-Vα24 (clone C15) and anti-Vβ11 (clone C21) mAb (27, 29, 60, 61). However, this mAb combination leads to overestimation of type I NKT cell numbers since conventional T cells can also express Vα24 and Vβ11 TCR subunits (62, 63). Alternatively, type I NKT cells can be detected with anti-Vα24Jα18 (Clone 6B11) mAb (28, 31). Furthermore, α-GalCer-loaded CD1d dimers (64) and tetramers (30, 65–67) can be used to specifically detect CD1d-restricted type I NKT cells, for instance, in combination with anti-CD3, anti-Vα24, or anti-Vβ11 mAb. Importantly, Sag et al. reported on the detection of cytokines in type I NKT cells upon stimulation, which enables accurate identification of different functional NKT cell subsets in future studies (68). Since this approach has not been used in NKT phenotype studies yet, no information is available on the phenotype of the different functional NKT cell subsets.

### Phenotype of Type I NKT Cell Subsets

Type I NKT cells constitutively express various T cell markers such as the TCR signaling complex CD3, and costimulatory receptors such as CD4, CD8, and CD28 (27–31). CD4 is expressed by 15–80% of the type I NKT cell population (27–29, 31) and is sometimes used to subdivide type I NKT cells into CD4^−^ and CD4^+^ populations. Besides, type I NKT cells constitutively express various receptors that are usually observed on NK cells, such as the adhesion molecule CD56 and the activating NKR CD161 (27–30). 17–70% of the CD4^−^ type I NKT cells express CD56, in contrast to only a small fraction of the CD4^+^ type I NKT cell population (3–11%) (29). Interestingly, type I NKT cells acquire a memory-activated phenotype before birth (unlike NK- and T cells), reflected by high expression of CD45RO and low expression of the homing receptor CD62L (28, 29, 31, 69). This might indicate that these NKT cells have been sensitized and activated during fetal life by encountering a natural ligand (69), which contributes to the ability of NKT cells to respond fast upon meeting the antigen.

In addition, type I NKT cells have the ability to induce expression of a number of phenotypic markers upon activation and/or interactions within the microenvironment. For instance, type I NKT cells can upregulate CD62L expression upon α-GalCer-mediated activation and expansion which can serve as a marker for NKT cells with superior survival and proliferative capacity (70). In addition, CD4^+^ and DN type I NKT cells express CD69, which is involved in lymphocyte proliferation (28, 71). Upon cytokine-mediated activation, type I NKT cells upregulate CD69 expression. Furthermore, CD4^+^, CD8^+^, and DN type I NKT cells express CD27, a costimulatory immune-checkpoint molecule involved in the control of T cell immunity (28, 29, 31). Remarkably, expression of CD27 seems to be downregulated on type I NKT cells upon activation with α-GalCer, whereas its expression is upregulated in activated T cells (72, 73). This downregulation could be related to the fact that NKT cells already constitute a memory phenotype and, therefore, do not require CD27 to generate NKT cell immunity and maturation upon first antigen encounter. α-GalCer-activated type I NKT cells also express the costimulatory molecule CD40L, and the activation marker CD38 (28, 30, 31, 71). In addition, type I NKT cells express the inhibitory NKR NKG2A, the low affinity Fc receptor CD16, and the activating NKRs DNAM-1, NKG2D, NKp30, NKp44, NKp46, and 2B4, that are usually expressed by NK cells (27–31). The proportion of type I NKT cells expressing specific NKRs is highly variable among healthy individuals (1–85%) and can be altered upon interactions within the TME (31, 56, 74–78). In addition to the expression of NK- and T cell-associated cell surface markers, type I NKT cells express a wide range of inducible cytokine- and chemokine receptors enabling them to respond to various signals (28–31, 79–81). For instance, type I NKT cells induce expression of the IL-2 receptor chain CD25 (IL-2RA) upon α-GalCer-mediated activation (28, 29, 31), primarily in the CD4^+^ type I NKT cell population (30). A different pattern is observed regarding the chemokine receptors CCR5, CCR6, CCR7, and CXCR6, which are all higher expressed on CD4^−^ type I NKT cells compared with CD4^+^ type I NKT cells (27, 29, 30). Finally, type I NKT cells express various markers that are involved in a wide range of functionalities such as granzyme B, perforin, and CD95L, which play important roles in cytotoxicity (29, 31, 82).

### Functional Type II NKT Cell Subsets

So far, two distinct functional type II NKT cell subsets have been identified (Figure 1). T_H_1-like type II NKT cells secrete the pro-inflammatory cytokines IFN-γ and TNF-α upon stimulation, whereas T_H_2-like type II NKT cells secrete the regulatory cytokines IL-4 and IL-13 (19, 83–85). Murine studies showed that the cytokine profile of type II NKT cells can be influenced in the same way as has been observed for type I NKT cells (83), suggesting plasticity of type II NKT cell subsets as well.

### Identification of Type II NKT Cell Subsets

In contrast to type I NKT cells, no specific methods exist to identify the entire type II NKT cell population due to the lack of specific markers. However several mouse models have been developed to study the role of type II NKT cells in cancer *in vivo*. These models include Jα18^−/−^ mice, without type I NKT cells, and CD1d^−/−^ mice that lack both type I and type II NKT cells (86–88). Another approach to study type II NKT cells in both mice and humans is by using sulfatide-loaded CD1d multimers (19, 84, 89, 90). However, this approach has not been widely used due to the unstable nature of sulfatide-loaded CD1d complexes. Furthermore, since not all type II NKT cells are sulfatide reactive, this method excludes a significant proportion of type II NKT cells (83, 89). As a result, the phenotype and function of type II NKT cells remain largely elusive, and new methods are essential to characterize this cell population in further detail.

### NKT-Like Cells

In many studies, NKT cells are identified with flow cytometry using a combination of anti-CD3 and anti-CD56 mAb (31, 81, 91–94). Although it is likely that the CD3^+^CD56^+^ cell population includes CD1d-restricted NKT cells, it has to be taken into consideration that conventional T cells have been reported to express NK-cell markers as well, including CD56 (17, 31, 79, 80). Since it is unclear whether CD3^+^CD56^+^ cells are CD1d restricted, this population is often referred to as “NKT-like.” An additional marker is essential to determine which part of the NKT-like cells are true NKT cells and which are not. Besides, only a small part of the type I NKT cell population expresses CD56 (29). Hence, a significant proportion of type I NKT cells is excluded from analyses when using the combination of anti-CD3 and anti-CD56 mAb. NKT-like cells express costimulatory-, cytokine-, and chemokine receptors, and NKRs that are also expressed by type I NKT cells (27–31, 81). Exceptions are killer-cell Ig-like receptors (KIRs) that provide either inhibitory or stimulatory signals upon interaction with human leukocyte antigen (HLA) molecules (31, 95, 96). KIRs are primarily expressed by NKT-like cells, and not by type I NKT cells.

In conclusion, different functional NKT cell subsets can be identified within the type I and type II NKT cell populations. Although type I NKT cells are characterized in detail, type II NKT cells are not due to lack of specific markers. Studies so far suggest that the expression levels of cell surface markers on type I NKT cells are highly variable among healthy individuals. Interestingly, murine studies showed that expression patterns of type I NKT cell surface markers are modulated upon cell–cell interaction and/or interaction with signaling molecules (74, 76). These phenotypical modulations adapt the functional capabilities of the NKT cells, including the production of specific cytokines upon activation. These data indicate a high degree of NKT cell plasticity and that type I NKT cells (and probably type II NKT cells as well) are able to switch between different phenotypical/functional subsets. Studies on human NKT cells are needed to support this hypothesis.

## Activation of NKT Cells

Due to expression of both NK- and T cell-associated functional molecules, NKT cells can be activated by mechanisms utilized by both NK- and T cells.

### Activation *via* T Cell-Like Mechanisms

**Figure 2:**
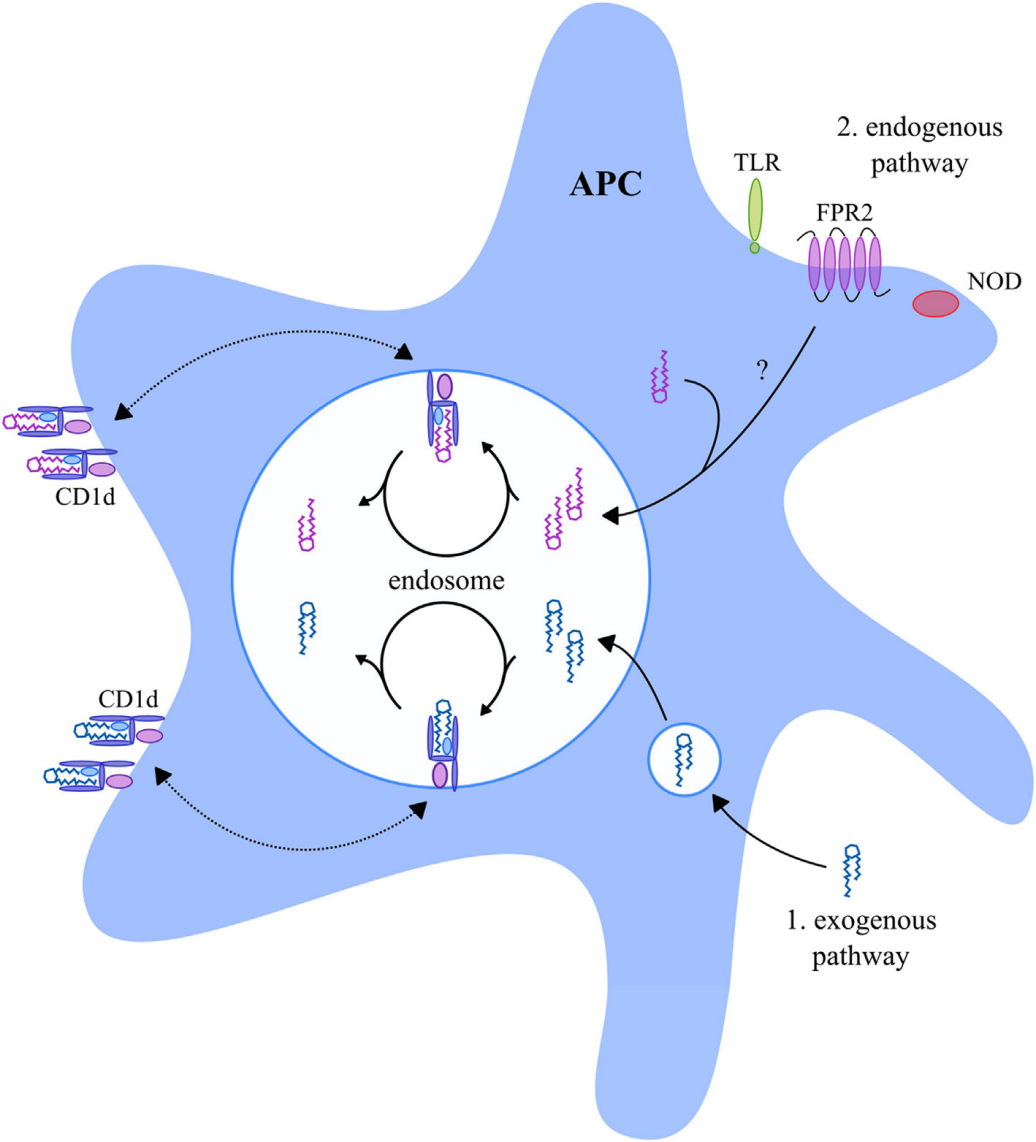
CD1d-presented glycolipids in APC. APC present exogenous and endogenous glycolipids in the context of CD1d. During endosomal trafficking, CD1d molecules relocate from the cell membrane toward a late endosome where the bound glycolipid antigens are removed from CD1d and replaced by new glycolipid antigens. Exogenous glycolipids (indicated in blue) enter the APC late endosome *via* endocytosis or phagocytosis (1). Endogenous glycolipids (indicated in purple) enter the late endosome as a result of NOD, FPR2, or TLR signaling (2), but the exact mechanism is unknown. The exogenous and endogenous glycolipids are loaded into CD1d molecules upon which they relocate back to the cell membrane. Abbreviations: APC, antigen-presenting cells; NOD, nucleotide-binding oligomerization domain; FPR2, formyl peptide receptor 2; TLR, toll-like receptor.

First, NKT cells can be activated *via* their TCR in a T cell-like manner *via* recognition of glycolipids in the context of CD1d molecules (61, 97). CD1d is primarily expressed by antigen-presenting cells (APC) but has also been reported to be expressed by some epithelial, parenchymal, and vascular smooth muscle cells (98, 99). Importantly, APC are able to present both exogenous and endogenous glycolipids in the context of CD1d (Figure 2). Exogenous microbial- and non-microbial-derived glycolipids enter APC *via* different mechanisms as thoroughly reviewed by Bendelac et al. (100) and Barral and Brenner (101). For instance, exogenous glycolipids can be captured by the mannose receptor, or alternatively, insert themselves directly into the cell membrane of APC, upon which they undergo endocytosis. Furthermore, exogenous glycolipids may enter APC with very low-density lipoprotein particles *via* the low-density lipoprotein (LDL) receptor, or *via* phagocytosis. Finally, scavenger receptors can mediate internalization of apoptotic cells and modified LDL, which also leads to entering of exogenous glycolipids into APC. During endosomal trafficking, CD1d molecules relocate from the cell membrane toward a late endosome where the bound glycolipids are removed from CD1d and replaced by new glycolipids (Figure 2) (102, 103). Thereafter, the CD1d molecules relocate back to the cell membrane. In addition, APC also present endogenous glycolipids in the context of CD1d (Figure 2). For instance, activation of nucleotide-binding oligomerization domain-1 and -2 intracellular pattern recognition receptors by bacteria, or activation of formyl peptide receptor 2 by serum amyloid A-1, results in loading of endogenous glycolipids into CD1d molecules during endosomal CD1d trafficking (Figure 2) (104, 105). Furthermore, toll-like receptor signaling upon stimulation with lipopolysaccharide was suggested to result in the loading of endogenous glycolipids into CD1d molecules (100, 106). The exact mechanism of how these signaling pathways lead to the loading of endogenous glycolipids into CD1d is, however, unknown.

In contrast to TCRs on conventional T cells that recognize specific peptides presented by MHC class I or II, the Vα24Jα18 TCR present on type I NKT cells recognizes a diversity of glycolipids that are presented by CD1d molecules. For instance, type I NKT cells recognize glycosphingolipids, α-galactosyldiacylglycerols, diacylglycerols, and phospholipids derived from mycobacteria in addition to α-GalCer (107–111). Furthermore, type I NKT cells can be activated upon encountering the self-glycolipids isoglobotrihexosylceramide and β-glucosylceramide (11, 112). In addition, type II NKT cells have been reported to recognize the self-glycolipids sulfatide and β-glucopyranosylceramide, as well as lysophospholipids and microbial lipids (19, 83, 89, 113, 114). As a consequence of their diverse TCR repertoire, different type II NKT cell subsets exist, recognizing different lipids (19, 85).

Activated NKT cells are able to kill tumor cells directly in a CD1d-dependent manner (115). This antigen-specific cytotoxicity is CD95/CD95L dependent, unlike NK- and T cells that predominantly use perforin/granzyme-mediated mechanisms (115). Upon activation *via* their TCR in a CD1d-dependent manner, NKT cells rapidly expand and secrete a range of cytokines (68, 116–120). Crowe et al. reported ~10-fold expansion of type I NKT cell numbers in the murine spleen 2–3 days after injection with 2 µg α-GalCer in mice (116). Besides, ~7- and ~3-fold type I NKT cell expansion was reported in the liver and bone marrow of mice 2–3 days after α-GalCer injection, respectively (116). Due to their memory-activated phenotype (69), NKT cells have the ability to respond quickly upon encountering an antigen. Within an hour after injection with α-GalCer, a burst of cytokines can be detected in mice. For instance, studies reported maximal levels of IFN-γ^+^ and IL-4^+^ murine liver- and splenic-derived type I NKT cells within 2 h after α-GalCer activation *in vivo* (116, 117). In addition, high IFN-γ (400 pg/ml) and IL-4 (1,500 pg/ml) levels were detected in the serum of these mice 90 min after injection with 100 ng/ml α-GalCer (117). Although the percentage of IL-4^+^ splenic-derived type I NKT cells dropped to baseline levels 16 h after injection of mice with 2 µg α-GalCer, elevated IFN-γ^+^ type I NKT cells could still be detected after 72 h (116). In conclusion, NKT cells rapidly secrete a range of cytokines following activation with α-GalCer.

### Activation *via* NK Cell-Like Mechanisms

**Figure 3:**
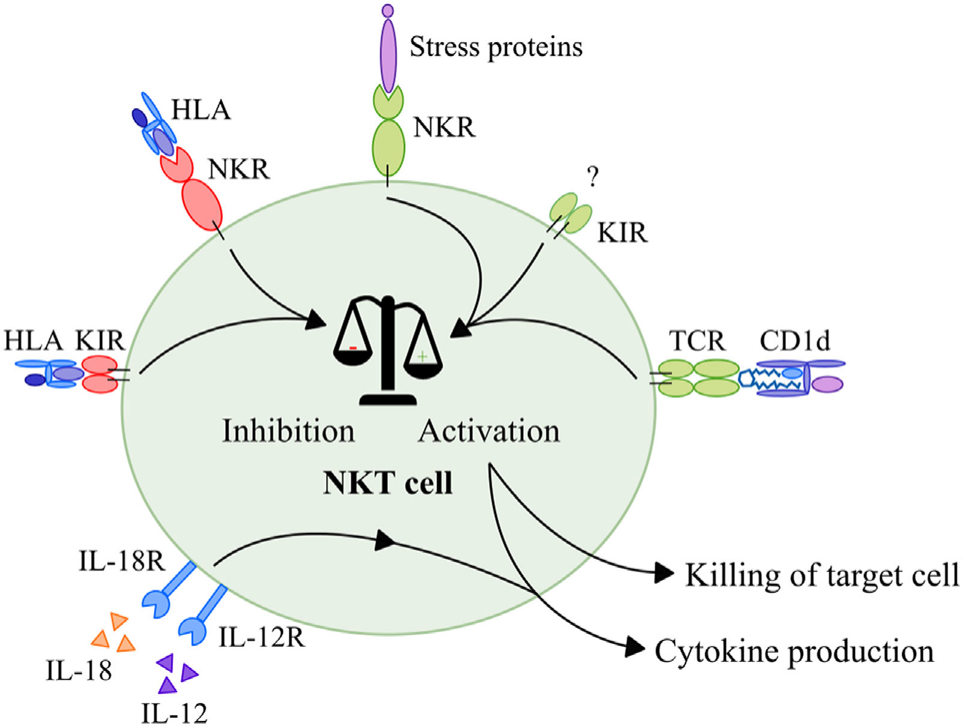
Activation of NKT cells. Activation of NKT cells is dependent on a balance between activating and inhibitory signals. First of all, NKT cells receive activating signals *via* their TCR that recognize glycolipids in context of CD1d. Second, NKT cells receive activating signals in a CD1d-independent manner *via* NKRs upon interaction with stress proteins such as MIC-A and MIC-B, ULBPs, Nectin-2, or MLL5 proteins. In addition, NKT cells receive activating signals in a CD1d-independent manner *via* KIRs with merely unknown ligands. Inhibitory NKRs and KIRs that recognize HLA molecules provide inhibitory signals that are able to disrupt both TCR and NKR signaling. When the balance is skewed toward activation, NKT cells produce and secrete high amounts of cytokines. In addition, NKT cells that are activated *via* this mechanism are able to kill target cells directly *via* activation of their TCRs and NKRs. Finally, NKT cells—like NK cells—can be activated by a combination of IL-12 and IL-18 which leads to production of cytokines. Abbreviations: NKT, natural killer T; TCR, T cell receptor; NKR, natural killer receptor; MIC, major histocompatibility complex I-like molecules; ULBP, unique long-binding protein; MLL5, mixed-lineage leukemia-5; KIR, killer-cell Ig-like receptor; HLA, human leukocyte antigen; NK, natural killer.

NKT cells seem to behave similar to NK cells when it comes to their activation. Like in NK cells, activation is dependent on the balance between inhibitory and stimulatory signals obtained *via* NKRs and KIRs (121, 122). As discussed earlier, phenotype studies showed that NKT cells also express a wide range of these receptors (27–31). Activating NKRs are able to recognize a variety of MHC-like molecules and cellular targets often referred to as “stress proteins.” For instance, the NKG2D receptor recognizes MHC class I-like molecules (MIC) A and B and unique long-binding proteins (123), whereas DNAM-1 recognizes the poliovirus receptor and Nectin-2 (124). Besides, NKT cells express KIRs, with less well-defined ligands, that provide activating signals (125–128). Furthermore, NKT cells express NKRs and KIRs that provide inhibitory signals upon binding with HLA molecules (129–134). When the balance of signals is shifted toward activation, an NKT cell is activated, resulting in cytokine production as well as direct killing of tumor cells in a CD1d-independent manner (Figure 3). Interestingly, studies showed that inhibitory signals provided by KIRs and/or NKRs were able to interrupt TCR signaling in conventional T cells without CD1d restriction (31, 130, 131, 133). Since NKT cells express similar functional receptors, it is likely that similar interruption of TCR signaling also occurs in NKT cells (Figure 3). In addition, a part of the NKT cell population expresses the low affinity Fc receptor CD16 which is known to induce antibody-dependent cytotoxicity when present on NK cells (135). This phenomenon has, however, not been studied as yet in relation to NKT cells. In conclusion, NKT cells can be activated *via* different NK- and T cell-associated mechanisms that lead to immediate killing of tumor cells and secretion of large amounts of cytokines that have a major influence on the immune system.

Finally, studies showed that NKT cells—like NK cells—can also be activated by IL-12 in combination with IL-18 *via* cytokine receptors in a CD1d-independent manner (Figure 3) (136–138). These cytokines are secreted by, i.e., active macrophages or dendritic cells (DCs) (139–141). Upon interaction with IL-12 and IL-18, NKT cells secrete high amounts of IFN-γ, as also observed after CD1d-dependent and NKR-mediated activation (127, 136, 137, 142).

### NKT Cell Anergy

Importantly, murine studies have indicated that overstimulation and chronic activation of type I NKT cells with α-GalCer *via* TCR–CD1d interaction may result in NKT cell death and induction of anergy (116, 143–147). This resembles the response of conventional T cells upon activation in the TME in presence of coinhibitory stimuli or checkpoint molecules like programmed death-ligand 1 (PD-L1) (148, 149). Upon binding with α-GalCer in the context of CD1d, type I NKT cells downregulate their TCR and NKR expression and upregulate the inhibitory molecules programmed cell death protein 1 (PD-1) and B- and T-lymphocyte attenuator (144, 146, 150), resulting in hyporesponsiveness. Furthermore, chronic stimulation of type I NKT cells with α-GalCer *in vivo* resulted in activation-induced cell death (AICD) *via* upregulation of the death receptor CD95, thereby contributing to active depletion of type I NKT cells (151, 152). This is most likely a feedback mechanism used by NKT cells to prevent tissue damage. Anergy induced by α-GalCer also resulted in impaired proliferation and production of IFN-γ by type I NKT cells upon α-GalCer restimulation. By contrast, anergic type I NKT cells retained their capacity to produce T_H_2-associated cytokines (144). Moreover, pretreatment with α-GalCer skewed type I NKT cells toward a T_H_2 or T_reg_-like profile (153–156). α-GalCer pretreated type I NKT cells acquired characteristics of regulatory cells *in vivo*, including production and secretion of IL-10, which is known to induce and maintain an immunosuppressive TME (153–155). Chronic stimulation of NKT cells in the TME might therefore contribute to immune escape in cancer patients.

## The Regulatory Function of NKT Cells

**Figure 4:**
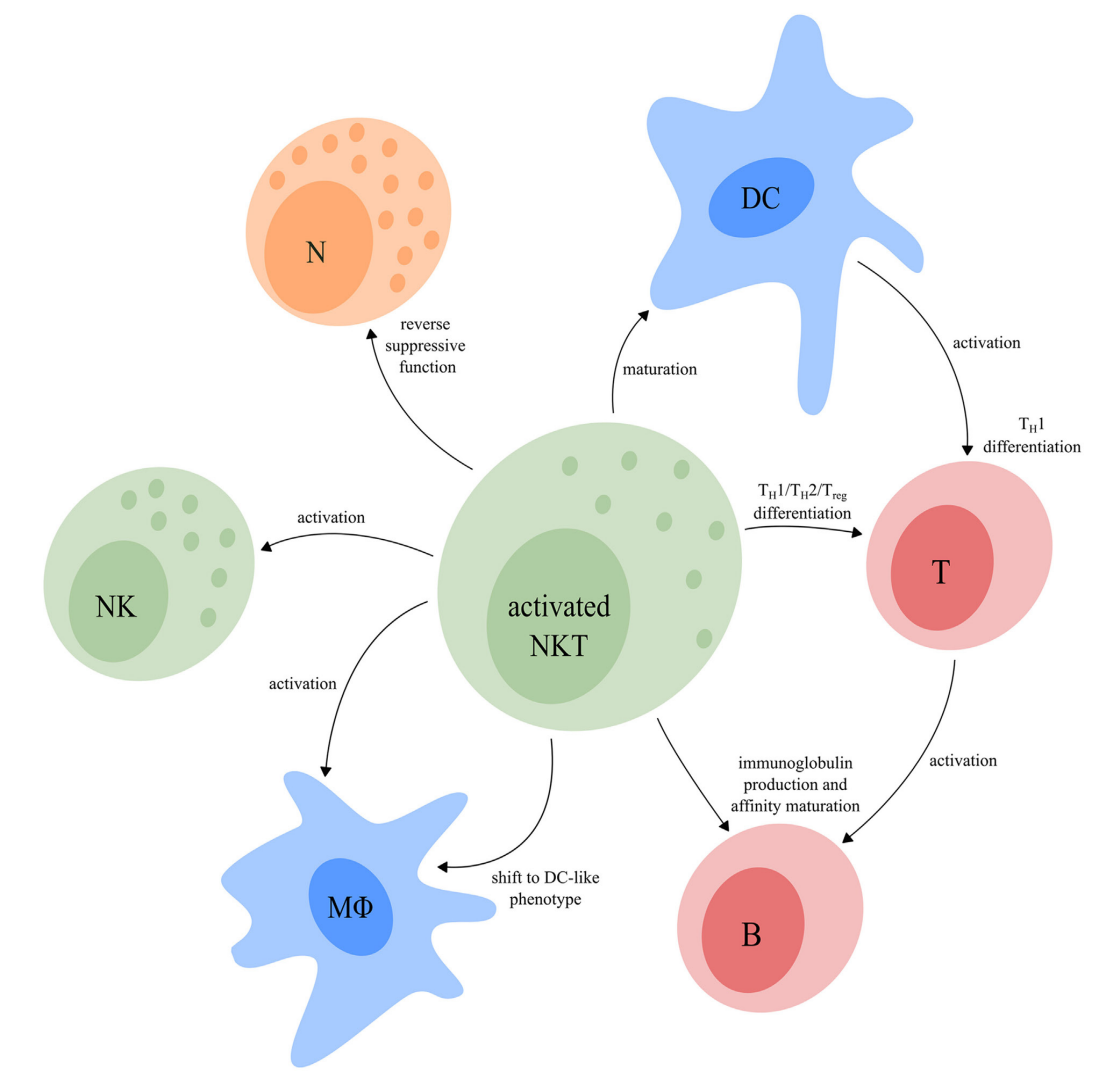
The regulatory function of activated NKT cells. Upon activation, NKT cells have a major influence on other immune cells. NKT cells can induce full maturation of DC upon which they activate T cells and induce T_H_1 differentiation. Activated T cells interact with B cells resulting in their activation, as well as production of immunoglobulins and affinity maturation. The T_FH_ type I NKT cell subset provides cognate help for B cells which promotes the production of immunoglobulins and affinity maturation. In addition, NKT cells can reverse the phenotype of immune suppressive neutrophils. Upon activation, NKT cells are also able to activate NK cells and macrophages and induce a functional shift of monocytes toward a DC-like phenotype. Finally, NKT cells can drive T cell differentiation to either a T_H_1-, T_H_2-, or T_reg_-profile, dependent on the NKT subsets involved. Abbreviations: NKT, natural killer T; DCs, dendritic cells; T_H_, helper T; T_FH_, follicular helper T; NK, natural killer; T_reg_, regulatory T; N, neutrophil; MΦ, macrophage.

Depending on which functional NKT cell subsets are involved, both type I and type II NKT cell subsets are able to either skew the immune response toward inflammation or toward tolerance. Activated NKT cells shape the TME *via* modulation of cells from both the innate and adaptive immune system (Figure 4), thereby implementing an important regulatory function.

NKT cells are unique in the sense that they can activate and induce full maturation of DC (139, 140, 157). This maturation requires direct interaction of DC with NKT cells *via* the TCR–CD1d complex in combination with CD40/CD40L costimulation. As a result, DC produce IL-12, which further drives IFN-γ production by T_H_1-like NKT cell subsets. By contrast, IL-13 and IL-4, produced by T_H_2-like NKT cell subsets, indirectly suppress T cell function and drive T_H_2 differentiation, respectively (49, 158–160). IL-10, produced by T_reg_-like type I NKT cells drives T cell differentiation toward T_regs_, thereby contributing to the establishment of an immunosuppressive TME (161). Finally, the IL-21 producing T_FH_-like type I NKT cell subset interacts directly with B cells that present the same glycolipid in context of CD1d as used to activate the NKT cells, resulting in fast immunoglobulin production and affinity maturation (53, 162–165).

In addition to production of large amounts of cytokines as discussed earlier, NKT cells secrete a range of chemokines upon activation, including RANTES, Eotaxin, MIP-1α, and MIP-1β, that lead to the attraction of NK cells, neutrophils, and monocytes toward the inflammatory microenvironment (166). IFN-γ secreted by T_H_1-like NKT cell subsets then leads to the local activation of NK cells, neutrophils, and macrophages (167–169). Furthermore, granulocyte macrophage colony-stimulating factor (GM-CSF), IFN-γ, and IL-4 secreted by NKT cells may shift the functional capacity of monocytes toward a more DC-like phenotype which contributes to the activation of T cells and, indirectly, B cells (170, 171). NKT cells are also able to reverse the phenotype of immune suppressive neutrophils by reducing secretion of IL-10 and enhancing IL-12 production in a CD1d-dependent manner (105).

In conclusion, NKT cells are able to rapidly respond to a wide variety of glycolipids and stress proteins using T- and NK cell-like mechanisms, respectively. Although NKT cells comprise a minor immune cell subset in most organs, they have a major effect on immune regulation since they can skew an immune response toward inflammation or tolerance in a very short time by secreting pro- or anti-inflammatory cytokines. Besides, NKT cells have the ability to kill tumor cells directly upon activation but, probably reflected by their relative low numbers, NKT cells primarily have a regulatory function. Based on this information, it is clear that NKT cells are not just cells with NK- and T cell properties: by combining characteristics of both cell types, NKT cells are able to add unique functions to the immune response. NKT cells may play a uniquely central role during the very first steps in the initiation of an antitumor immune response. The main reasons are the ability of NKT cells to respond fast by influencing other immune cells, resulting in amplification or dampening of the immune response.

## The *In Vitro* and *In Vivo* Antitumor Activity of NKT Cells

### Type I NKT Cells in Tumor Immunity

Twenty years ago, it was first reported that the glycolipid α-GalCer, discovered in marine sponges, had potent antitumor activity *in vivo* (172–174). Mice that were intravenously inoculated with B-16 (melanoma) or intraperitoneally inoculated with EL-4 (lymphoma) cells showed a significantly prolonged lifespan after injection with α-GalCer, with a stronger potency than the typical chemotherapeutic agent mitomycin C (172). A role for NKT cells in this antitumor activity was suggested a few years later when it was discovered that α-GalCer is recognized by type I NKT cells *via* their TCR in the context of CD1d, leading to their activation (175). Thereafter, studies showed that type I NKT cells were the key effectors of antitumor responses in a murine B-16 melanoma metastasis model (176–178). For instance, Toura et al. reported that activation of type I NKT cells *via* injection with α-GalCer-pulsed-DC resulted in complete eradication of established B-16 melanoma liver metastases (178). Administration of α-GalCer to activate type I NKT cells even prevented primary tumor formation in different *in vivo* models (179). By contrast, mice lacking type I NKT cells were more prone to chemical or p53 loss-induced tumor development (180–182). Recently, it was reported that type I NKT cells also play a role in preventing metastatic disease in a 4T1 mammary carcinoma model (183). Upon resection of the primary breast tumors, treatment with α-GalCer-pulsed-DC limited formation of tumor metastases, prolonged survival, and provided curative outcomes in ~45% of the mice. Thereafter, it was shown that α-GalCer-pulsed-DC could also be combined with the chemotherapeutics cyclophosphamide or gemcitabine to enhance survival of mice with metastatic disease (184). Importantly, studies showed that the anti-metastatic effect of α-GalCer was impaired in NK cell-depleted or IFN-γ-deficient mice (185, 186). Smyth et al. showed that IFN-γ production by type I NKT cells and subsequent IFN-γ production by NK cells was crucial for α-GalCer-mediated tumor protection (177). In line with these results, it was observed that the T_H_2-skewing synthetic α-GalCer-analog OCH provided less tumor protection in the CT26 mouse model compared with α-GalCer (187). By contrast, studies reported that analogs of α-GalCer, which skewed the cytokine profile of type I NKT cells toward T_H_1, provided superior protection against metastases formation compared with α-GalCer (188–190). This implicates a crucial role for type I NKT cells with a T_H_1 cytokine profile in antitumor activity.

After the discovery of the important role of activated type I NKT cells in antitumor responses *in vivo*, studies focused on the mechanisms used by these NKT cells to eradicate tumor cells. As mentioned earlier, CD1d is primarily expressed by APC, although malignant hematopoietic cells have also been reported to express CD1d on their cell membrane (4, 191–193). In addition, there is evidence that solid tumors also express CD1d, including renal cell and colorectal carcinomas (194, 195). Upon activation with α-GalCer, type I NKT cells were able to kill CD1d^+^ tumor cells in a CD1d-dependent manner (4, 191–193, 195). To kill tumor cells directly *via* CD1d interaction, they need to present glycolipids that can be recognized by NKT cells. There is evidence from murine studies that type I NKT cells can be activated by tumor-derived glycolipids that are cross-presented by APC in the context of CD1d (196–199). However, until now, the nature of tumor glycolipids that are recognized by NKT cells remains poorly elucidated. Since the cytolysis and eradication of tumor cells *via* type I NKT cells was shown to be dependent on CD1d expression on their cell surface, it was suggested that CD1d expression might be a predictor of whether α-GalCer-activated type I NKT cells are able to eradicate tumor cells or not (3, 4). However, other studies showed that CD1d^−^ hematopoietic cells could also be killed directly by type I NKT cells, for instance, *via* NKG2D activation (5, 200, 201). This illustrates that type I NKT cells, like NK cells, are able to kill tumor cells *via* NKR activation, even in the absence of CD1d. Moreover, although type I NKT cells are capable of killing tumor cells directly, they primarily mediate antitumor activity *via* the activation of downstream immune effector cells as demonstrated by human and mouse studies (4, 177, 202, 203). Especially T_H_1-like type I NKT cells play an important role in this antitumor activity *via* secretion of large amounts of IFN-γ, which leads to generation of tumor-specific CD8^+^ cytotoxic T cells, and rapid activation of NK cells (4, 177, 188, 202–204).

### Type II NKT Cells in Tumor Immunity

In contrast to type I NKT cells, only limited information is available regarding the role of type II NKT cells in cancer. However, some *in vivo* models provided important information. In general, type II NKT cells are associated with immunosuppression and tumor progression. For instance, murine carcinoma and lymphoma models showed that the tumor burden of CD1d^−/−^ mice, without type I and type II NKT cells, was lower compared with Jα18^−/−^ mice that lack type I NKT cells only (86–88). Injections with sulfatide increased the number of tumor nodules in a CT26 colon carcinoma lung metastasis mouse model *via* activation of type II NKT cells (205). Besides, administration of sulfatide abrogated the protective effect of α-GalCer-activated type I NKT cells against tumor development. Type II NKT cells were reported to produce IL-13 through the IL-4R–STAT6 pathway, which was necessary for downregulation of tumor immunosurveillance in a 15-12RM fibrosarcoma mouse model (49). Thereafter, it was shown that IL-13 induced TGF-β-secreting myeloid-derived suppressor cells (MDSCs) *in vivo* that inhibited tumor-specific T cells (158, 206). A role for MDSC in inhibition of antitumor immunosurveillance was supported by the study of Renukaradhya et al. that showed large numbers of these cells at the tumor site of B cell lymphoma-bearing Jα18^−/−^ mice without type I NKT cells (87). This implicates an important role for type II NKT cells in suppression of immunosurveillance in cancer. Although Zhao et al. showed in an *in vivo* murine model that activation of type II NKT cells with CpG oligodeoxynucleotides resulted in antitumor activity of these cells *via* the production of IFN-γ (83, 207). The involvement of IFN-γ implies that type II NKT cells are able to contribute to antitumor responses, but only when the T_H_1-like subset is involved.

In conclusion, a crucial role is implicated for type I NKT cells with a T_H_1 cytokine profile in antitumor activity. Although it is generally accepted that the type II NKT cell population promotes tumor growth, there is evidence that T_H_1-like type II NKT cells can be involved in antitumor responses. Hence, the role of NKT cells in malignancies is highly dependent on which functional type I or type II NKT cell subsets are involved.

## The Function and Phenotype of NKT Cells in Patients Diagnosed with Cancer

Several human studies have addressed the presence, function, and/or phenotype of NKT cells in cancer patients. Here, we will focus on both tumor-infiltrating and -circulating NKT cells.

### Tumor-Infiltrating NKT Cells

Studies showed a difference in the presence of NKT cells between tumor tissue and non-tumor tissue. The frequency of type I NKT cells was reported to be higher in intrahepatic malignant tumors and colorectal carcinomas compared with normal liver tissue and normal mucosa, respectively (208, 209). The opposite pattern was reported by Kenna et al. who showed a significantly lower presence of liver-infiltrating type I NKT cells in colorectal liver metastases compared with healthy liver tissue (35). In addition, several studies showed a correlation between infiltrating NKT cell numbers and clinical outcome. High numbers of tumor-infiltrating type I NKT cells correlated with a relatively good clinical outcome in patients diagnosed with colorectal cancer and neuroblastoma (209, 210). High density of NK/NKT cells was also associated with prolonged overall survival in periampullary adenocarcinoma (including pancreatic cancer) patients (211). Accordingly, absence of infiltrating type I NKT cells and low numbers of infiltrating NKT-like cells correlated with poor patient survival and disease progression in neuroblastoma and gastric cancer, respectively (91, 212).

The function and phenotype of infiltrating type I NKT cells was addressed in studies on hepatocellular carcinoma, colorectal cancer, and neuroblastoma (35, 209, 212). Lower expression of CD56 and CD161 was reported on infiltrating type I NKT cells in tumor-bearing livers compared with normal livers (35). In addition, in a study on colorectal cancer, expression of the activation markers CD69L and FasL was reported on a larger fraction of infiltrating type I NKT cells in tumor tissue compared with normal mucosa (209). Tumor-infiltrating type I NKT cells expressed IFN-γ and granzyme B, but the authors did not compare the expression of these markers to that of type I NKT cells in normal mucosa (209). In addition, it was observed that type I NKT cell infiltration in neuroblastomas was associated with CCL2 expression on tumor cells, indicating that expression of homing receptors on tumors was essential for infiltration of type I NKT cells in neuroblastoma (212). Furthermore, in two studies (91, 92) the function and phenotype of infiltrating NKT-like cells in tumors were described. Peng et al. reported impaired effector function of infiltrating NKT-like cells in gastric cancer-derived tumor tissue compared with non-tumor tissue, characterized by decreased expression of IFN-γ, TNF-α, granzyme B, and Ki-67 (91). Furthermore, this study also showed decreased expression of the lymphocyte proliferation marker CD69, the homing receptors CXCR3 and CCR5, and the NKRs NKG2D and DNAM-1 on NKT-like cells in tumor tissue compared with non-tumor tissue. In addition, a study on patients with hepatocellular carcinoma showed that NKT-like cells in tumor tissue expressed FOXP3 and lost expression of IFN-γ and perforin compared with NKT-like cells in non-tumor tissue (92).

In conclusion, tumor-infiltrating type I NKT cells and NKT-like cells may express less activating receptors, homing receptors and proliferation markers and produce lower amounts of T_H_1-associated cytokines compared with type I NKT cells and NKT-like cells in healthy tissue, indicating tolerance and not antitumor activity.

### Circulating NKT Cells

In addition, studies also showed altered function of circulating type I NKT cells in cancer patients. For instance, the number of circulating type I NKT cells was significantly decreased in patients with different cancers compared with healthy controls (213–221). In line with the results on infiltrating type I NKT cells, low circulating type I NKT cell numbers correlated with poor clinical outcome in patients with head and neck squamous cell carcinoma (214, 215). Interestingly, late-stage cancer patients presented with lower type I NKT cell numbers than early-stage cancer patients with oral squamous cell carcinoma or laryngeal cancer (218, 221), suggesting cancer-mediated depletion of NKT cells. After resection of the primary tumor, type I NKT cell numbers did not increase in patients with different cancer types (213, 220). By contrast, circulating NKT-like cell numbers were not decreased in patients diagnosed with laryngeal cancer, gastric cancer, or hepatocellular carcinoma (91, 92, 220).

Besides being reduced in numbers, circulating type I NKT cells are often functionally impaired in patients (216, 218, 219, 222–224). For instance, circulating type I NKT cells derived from patients with prostate cancer or oral squamous cell carcinoma had a T_H_2-biased cytokine profile (218, 219). Furthermore, type I NKT cells obtained from patients with advanced cancer stages showed impaired cytokine production and proliferative capacity upon *ex vivo* activation with α-GalCer (216, 219, 222). In accordance with this observation, lower numbers of IFN-γ-producing type I NKT cells were observed in patients with colon carcinoma, head and neck cancer, breast cancer, or renal cell carcinoma compared with healthy controls (213). These changes in cytokine profile imply that type I NKT cells switched from a T_H_1- toward a T_H_2-like NKT cell subset.

In conclusion, reduced frequency of circulating NKT cells and altered phenotype, resulting in altered function, of both infiltrating and circulating NKT cells are often observed in cancer patients, especially in patients with late-stage disease. Since altered function was not observed in infiltrating NKT cells in healthy tissue, it can be argued that this altered function of NKT cells is cancer/TME mediated: tumors may suppress the immune system, and skew the cytokine profile of NKT cells from T_H_1 toward T_H_2 to escape from recognition and elimination. Although the mechanisms behind the cancer/TME-mediated altered function of NKT cells are not fully understood, studies suggested a role for metabolic derivative lactic acid (225), the production of soluble factors by tumors such as sMIC (226), and the expression of CD1d by tumors (194, 227).

## Shaping the TME by NKT Cells

### Shaping the TME by T_H_1-Like NKT Subsets

**Figure 5:**
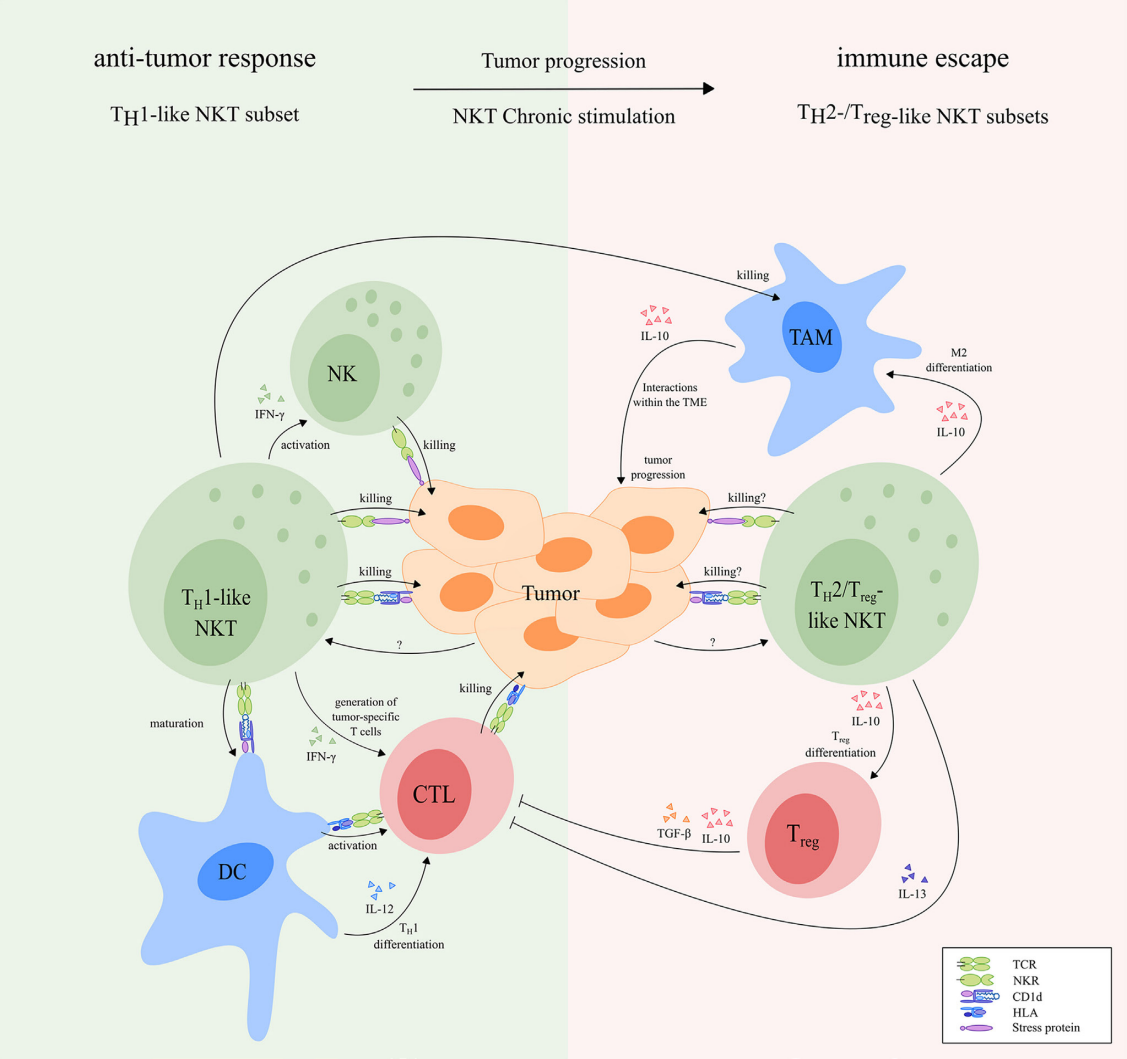
The dual role of NKT cells in cancer. During early tumor development, T_H_1-like NKT cells may induce an effective antitumor response *via* direct killing of tumor cells upon interaction with stress proteins and CD1d molecules, respectively. In addition, activated T_H_1-like NKT cells secrete high amounts of IFN-γ, which may lead to generation of tumor-specific CD8^+^ cytotoxic T cells and rapid activation of NK cells that kill tumor cells. Besides, activated NKT cells can induce maturation of DC in a CD1d-dependent manner, resulting in T_H_1 differentiation and activation of tumor-specific CD8^+^ T cells. Finally, activated T_H_1-like NKT cells can kill M2-polarized TAM in a CD1d-dependent manner, thereby preventing their tumor growth-promoting effects. During tumor progression, T_H_1-like NKT cells can become anergic and may switch to T_H_2-/T_reg_-like NKT cell subsets that facilitate tumor progression and immune escape. For instance, T_reg_-like NKT cells can promote differentiation of M2-polarized TAM and T_regs_. T_regs_ inhibit tumor-specific T cells *via* cell–cell interactions, and secretion of IL-10 and TGF-β. In addition, T_H_2-like NKT cells can inhibit tumor-specific T cells *via* production of large amounts of IL-13. T_H_2- and T_reg_-like NKT cell subsets might be able to kill tumor cells, either *via* CD1d-dependent or CD1d-independent mechanisms. However, overstimulated NKT cells produce large amounts of immunosuppressive cytokines, resulting in a net effect of immunosuppression. Receptors that are known to be involved in NKT cell-mediated antitumor responses are indicated in the figure. The functional NKT cell subsets indicated in the figure can be both type I and type II NKT cells as illustrated in Figure 1. Abbreviations: NKT, natural killer T; T_H_, helper T; NK, natural killer; DCs, dendritic cells; TAM, tumor-associated macrophages; T_reg_, regulatory T; TME, tumor microenvironment; NKR, natural killer receptor; TCR, T cell receptor; HLA, human leukocyte antigen.

As discussed earlier, T_H_1-like NKT cells are promising candidates to initiate effective antitumor immune responses. T_H_1-like NKT cells might play an important role in antitumor responses by shaping the TME (Figure 5). For instance, they have been reported to colocalize with tumor-associated macrophages (TAM) with an M2-polarized phenotype that promote tumor growth and progression (155, 228). This colocalization resulted in CD1d-dependent killing of TAM that cross-presented tumor-derived glycolipids *in vivo* (197, 229). Furthermore, T_H_1-like NKT cells and secondary activated NK cells contributed to the inhibition of tumor angiogenesis by IFN-γ *via* suppression of M2-polarized TAM (230, 231). Finally, Courtney et al. showed that type I NKT cells were able to kill CD1d^+^ M2 TAM or polarize M2 TAM toward an M1-polarized phenotype *via* GM-CSF production (232). Hence, the presence of T_H_1-like type I NKT cells might minimize the presence of tumor growth-promoting M2-polarized TAM in the TME. On the other side, an immature tolerogenic DC subset has been described that produces reduced amounts of IL-12 and high amounts of IL-10, resulting in an immunosuppressive TME (233). As a result, tolerogenic DC skew differentiation of naïve T cells into T_regs_, which might lead to immune escape of tumor cells (234–236). Since T_H_1-like NKT cells are able to fully mature DC, the presence of immature tolerogenic DC might be minimized in tumors where sufficient numbers of these NKT cells are present. Importantly, T_H_1-like NKT cells are able to stimulate both tumor antigen-restricted T cells that recognize tumor cells with HLA expression and effector NK cells that eliminate tumor cells with low or absent HLA expression (234). In this way, immune escape of tumor cells might be prevented. In addition, as discussed earlier, tumors have been reported to express CD1d on their cell membrane (194, 195) and might therefore be killed in a CD1d-dependent manner. Besides, tumors are reported to express high cell surface densities of stress-related proteins that activate the NKRs NKG2D and DNAM-1 (237, 238), suggesting that these cells can also be killed in a CD1d-independent manner. HLA class I loss or downregulation has often been reported in tumors including carcinomas, sarcomas, neuroblastomas, and melanomas (239–242). Since inhibitory NKRs and KIRs prevent NKT cell activation upon interaction with HLA molecules (Figure 3), NKT cells might be able to directly kill tumor cells with low HLA expression, similar to NK cells. NKT cells may have, however, primarily a regulatory function, suggesting that the antitumor activity mediated by direct killing of tumor cells is of lesser importance.

### Shaping the TME by T_H_2-/T_reg_-Like NKT Subsets

As discussed in the previous chapter, the phenotype and function of T_H_1-like subsets is frequently altered in patients. The NKT cell population in patients is skewed toward a T_H_2 profile, proliferative impaired and, in addition, reduced in size. These data indicate many similarities with overstimulated/anergic NKT cells. During cancer progression, NKT cells may be exposed to chronic stimulation, which is known to induce anergy and skew NKT cells toward immunosuppressive subsets. Moreover, chronic stimulation of NKT cells activates AICD, which might explain the reduced NKT cell population observed in cancer patients. Based on this hypothesis, we propose that T_H_1-like NKT cells induce an effective antitumor response during early tumor development and perhaps prevent further tumor development in many cases. However, in some cases, at some point during tumor progression, NKT cells become overstimulated. As a result, a part of the NKT cell population is deleted in cancer patients *via* AICD. In addition, the remaining NKT cells become hyporesponsive, or switch to T_H_2-/T_reg_-like NKT cell subsets, thereby facilitating tumor progression and immune escape (Figure 5). T_H_2- and T_reg_-like NKT cell subsets do not produce IFN-γ which is responsible for most of the antitumor effects of T_H_1-like NKT cells as discussed earlier. By contrast, T_H_2-/T_reg_-like NKT cells produce large amounts of IL-13 and IL-10, respectively, that suppress the TME (i.e., *via* fibroblasts and MDSC), thereby indirectly stimulating tumor progression. In addition, T_reg_-like NKT cells promote differentiation of M2-polarized TAM and T_regs_ that are also able to suppress the TME *via* production of IL-10 (154, 155). In contrast to T_H_1-like NKT cells, T_H_2-like NKT cells are not capable of inducing DC maturation and do therefore not induce activation of tumor-specific T cells (157). T_H_2-like NKT cell subsets further inhibit the activation of tumor-specific T cells *via* secretion of IL-13 (49, 158, 159), while T_regs_ inhibit tumor-specific T cells *via* cell–cell interactions and secretion of IL-10 and TGF-β (243, 244). T_H_2- and T_reg_-like NKT cell subsets might still be able to kill tumor cells, either *via* CD1d-dependent or CD1d-independent mechanisms. However, overstimulated NKT cells produce large amounts of immunosuppressive cytokines, resulting in a net effect of immunosuppression. T_H_2- and T_reg_-like NKT cell subsets, therefore, counteract the antitumor effects of T_H_1-like NKT cells and, in addition, actively promote tumor progression.

In conclusion, we discussed evidence supporting our hypothetical model (Figure 5) that T_H_1-like NKT cells are responsible for initiating effective antitumor immune responses during early tumor development. When NKT cells become overstimulated and anergic due to tumor progression, a part of the NKT cell population is deleted in cancer patients. In addition, the remaining NKT cells lose their antitumor function and start facilitating immune escape and tumor progression. In summary, we illustrated three problems regarding NKT cells in cancer patients. First, the numbers are lower compared with healthy individuals. Second, NKT cells are often anergic in cancer patients. Third, NKT cells are often skewed toward immunosuppressive T_H_2-like subsets.

## Current NKT Cell-Based Immunotherapy for the Treatment of Cancer

Because of their potential to induce effective antitumor responses *in vivo*, several NKT cell-based immunotherapies in humans have been developed over the past years as thoroughly reviewed by Nair and Dhodapkar (245). These immunotherapies primarily focused on activation and expansion of the type I NKT cell population.

For instance, a phase I clinical trial was executed in which intravenous (i.v.) injections of 50–4,800 µg/m^2^ α-GalCer were administered to 24 patients with different solid tumors (217). The majority of patients presented with reduced numbers of type I NKT cells at baseline (median 333 cells/ml PB) compared with healthy donors (median 1,013 cells/ml PB). Following α-GalCer administration, NKT cells disappeared from the circulation within 24 h. Although not investigated in details in this study, recovery of NKT cell numbers was not observed within a week. Furthermore, increased serum levels of TNF-α and GM-CSF were detected in five patients and increased serum IFN-γ levels were detected in one patient after α-GalCer administration. A phase II trial using administration of α-GalCer was not executed. In addition, clinical trials were conducted in which patients were injected with α-GalCer-pulsed autologous DC (246–252). These injections resulted in expansion of the type I NKT cell population in some patients (246–252). For instance, Chang et al. reported >100-fold expansion of type I NKT cells in 5 patients upon i.v. injections with 5 × 10^6^ α-GalCer-pulsed DC (247). Besides NKT cell expansion, treatment with α-GalCer-pulsed DC also increased the systemic levels of IFN-γ in patients (246–252). Treatment did, however, not result in a clinical tumor response in the majority of patients (246–252). In addition, a phase II study reported stable disease in 5 of 17 patients with non-small cell lung cancer (NSCL) upon i.v. administration of α-GalCer-pulsed IL-2/GM-CSF-cultured PBMC (1 × 10^9^ cells/m^2^) (251). Patients with increased IFN-γ producing T_H_1-like type I NKT cells after treatment showed a prolonged median survival time compared with non-responsive patients. These data indeed indicate a crucial role for T_H_1-like type I NKT cells in antitumor immune responses and emphasize the essential need for expansion of this NKT cell population in cancer patients. Hence, immunotherapeutic approaches focused on skewing NKT cells toward a T_H_1 profile should be developed.

In later clinical trials, *ex vivo*-activated type I NKT cells were adoptively transferred to patients diagnosed with NSCL, advanced melanoma, or head and neck squamous cell carcinoma, in some cases in combination with α-GalCer-pulsed APC (253–256). In this therapy, PBMC obtained from the patient, i.e., by leukapheresis, were cultured in the presence of IL-2 and α-GalCer to facilitate proliferation and activation of the type I NKT cell population. Thereafter, the *ex vivo*-activated type I NKT cells were administered to the patients. Phase I and II clinical trials were conducted in which patients with head and neck carcinomas received nasal submucosal injections of 1 × 10^8^ α-GalCer-pulsed APC, in combination with intra-arterial infusion of 5 × 10^7^ *ex vivo*-activated autologous type I NKT cells *via* tumor-feeding arteries (255, 256). Tumor regression and stable disease were reported in 10 of 10 of these patients (255). These clinical responses did, however, not correlate with the induction of immunological responses in blood (i.e., increase in type I NKT cell numbers and/or IFN-γ-producing type I NKT cells and NK cells). In addition, *ex vivo*-activated type I NKT cells were adoptively transferred to patients diagnosed with advanced or recurrent NSCL (1 × 10^7^ or 5 × 10^7^/m^2^ NKT cells per infusion) or advanced melanoma (~4 × 10^6^–~2 × 10^8^ NKT cells per infusion) in phase I clinical trials (253, 254). Treatment was well tolerated and resulted in stable disease in 2 of 9 NSCL patients and 3 of 9 patients with advanced melanoma, respectively. However, the majority of patients developed progressive disease. This might be due to the fact that the numbers of administered *ex vivo*-activated autologous type I NKT cells were too low in comparison to the tumor load. Obtaining sufficient numbers of type I NKT cells might be a major challenge since type I NKT cell numbers are low in general, and especially in patients with cancer.

Recently, studies focused on increasing the specificity of NKT cells by transducing them with chimeric antigen receptors that are not HLA or CD1d restricted (58, 70, 257). In addition, α-GalCer/CD1d-antitumor fusion proteins were suggested as a treatment for cancer patients. For instance, α-GalCer-loaded CD1d molecules fused with an antibody fragment specific for HER2 or CEA antigens induced potent antitumor activity *in vitro* and *in vivo* (258, 259). Recently, Horn et al. showed that CD3 × PD-L1 Bi-specific T cell engagers activated both T cells and NKT cells to kill PD-L1^+^ tumor cells *in vitro* (260). Another strategy that was suggested for the treatment of patients with solid tumors is vaccination with NKT-activating agents in combination with tumor antigens. For instance, a phase I study showed detectable NKT cell activity in patients with high-risk melanoma upon treatment with cancer/testis antigen-loaded DC in combination with α-GalCer (261). However, in our opinion, increasing the specificity of NKT cells is not the most promising method of increasing the effectiveness of NKT-based immunotherapies. The strength of NKT cells does not rest in their cytotoxic capacities, but in their regulatory function. When the appropriate subsets are activated (i.e., T_H_1-like NKT cells), NKT cells might shift the tolerogenic and immunosuppressive state of both innate and adaptive cells toward antitumor activity. Therefore, instead of increasing the specificity of NKT cells, immunotherapies should focus on the most important function of NKT cells, their regulatory function.

In conclusion, several NKT cell-based immunotherapies have been tested in clinical trials. To date, a beneficial effect in a minority of cancer patients has been reported. These clinical trials were mainly based on the activation and expansion of type I NKT cells with α-GalCer. As addressed in our hypothetical model (Figure 5), NKT cells may switch to immunosuppressive functional subsets or become anergic due to chronic stimulation during cancer progression which might explain the absence of beneficial clinical responses in patients upon treatment with α-GalCer. We propose that it is essential to prevent and break NKT cell anergy in cancer patients and skew NKT cells in cancer patients toward T_H_1-like subsets with antitumor activity in addition to expansion of the NKT cell population.

## Focus of Future NKT Cell-Based Immunotherapies

In this review, we discussed the role of NKT cells in cancer and conclude that NKT cells play a central role in anticancer treatment due to their important regulatory function. To improve NKT cell-based immunotherapies for the treatment of cancer patients, several aspects of the current treatment strategies need further attention.

### Expansion of the NKT Cell Population

T_H_1-like NKT cells (either type I or type II) have the potential to induce effective antitumor responses. Combined with the fact that their numbers are decreased in cancer patients, it is essential to expand this cell population in patients. For instance, induced pluripotent stem cells might be used to expand the numbers of autologous NKT cells in patients *ex vivo* (262–264). Furthermore, culturing methods aiming at obtaining high numbers of NKT cells must be optimized. At the moment, according to the Clinical Trials registry, multiple clinical trials^1^ are ongoing that study the safety and clinical efficacy of adoptive type I NKT cell transfer in patients with solid tumors. As discussed in this review, this infusion should be accompanied by a protocol that prevents induction of NKT cell anergy and generation of immunosuppressive NKT cell subsets.

### Prevention and Breaking of NKT Cell Anergy

Until now, only a limited number of studies focused on prevention or breaking of NKT cell anergy. Parekh et al. showed that blockade of the interaction between PD-1 and its ligands prevented the induction of type I NKT cell anergy *in vivo* (265). Blockade of the PD-1/PD-L1 axis was, however, unable to reverse established NKT cell anergy (265, 266). In addition, *in vitro* and *in vivo* studies showed that stimulation of type I NKT cells with IL-2 overcomes anergy and restores their capacity to proliferate (144, 146). The proliferative capacity of patient-derived type I NKT cells was also reported to increase upon COX-2 inhibition or culture with G-CSF (222, 267). It might, therefore, be an option to treat patients with a combination of anti-PD-1 antibody, such as nivolumab, combined with IL-2/G-CSF or COX-2 inhibition to prevent and reverse NKT cell anergy.

### Skewing of NKT Cells toward T_H_1-Like Subsets

NKT cells in which anergy was reversed retained their T_H_2-biased cytokine profile upon IL-2 stimulation and did not change back toward a T_H_1-like subset with antitumor activity (146). It is therefore also necessary to use agents that are able to skew the cytokine profile of activated NKT cells toward a T_H_1 profile, which means a change in functional subset. For instance, culturing of patient-derived T_H_2-biased type I NKT cells with IL-12 resulted in IFN-γ production of these cells in response to α-GalCer *in vitro* (219). In addition, Laurent et al. showed that chemical modification of the α-GalCer compound was able to increase T_H_1-associated cytokine production by activated type I NKT cells, whereas stimulation of type I NKT cells with conventional α-GalCer resulted in production of both T_H_1- and T_H_2-associated cytokines (268). Other synthetic agonists have also been described that induce a T_H_1-skewed cytokine profile in type I or type II NKT cells (83, 190, 269, 270). Hence, the use of modified NKT cell-activating agents in cancer patients might skew the cytokine profile of NKT cells toward a T_H_1 profile while simultaneously preventing the induction of anergy.

## Concluding Remarks

Due to their important regulatory function, NKT cells are promising candidates for immunotherapies in patients diagnosed with cancer. However, NKT cell-based immunotherapies that focus on activating NKT cells have resulted in beneficial clinical responses in a minority of patients so far. In this review, we illustrated a hypothetical model regarding the role of NKT cells in solid tumors based on their function and phenotype. During early tumor development, T_H_1-like NKT cell subsets have the potential to initiate effective antitumor immune responses against tumors. However, when NKT cells become overstimulated and anergic during tumor progression, they lose their antitumor function and start facilitating immune escape. The role of NKT cells in cancer might therefore be more dynamic than initially thought. So far, studies have primarily focused on methods to activate and expand the type I NKT cell population in patients, but the contribution of functionally altered NKT cells to the failure of NKT cell-based immunotherapies has been largely ignored. In this review, we conclude that there should be three important focuses of future research in cancer patients: (1) expansion of the NKT cell population, (2) prevention and breaking of NKT cell anergy, and (3) skewing of NKT cells toward T_H_1-like subsets with antitumor activity.

## Author Contributions

DK compiled the literature sources, conceived of the presented idea, and wrote the manuscript. PK and MH contributed to scientific discussions and critically reviewed the manuscript.

## Conflict of Interest Statement

The authors declare that the research was conducted in the absence of any commercial or financial relationships that could be construed as a potential conflict of interest.

## Footnotes

^1^https://clinicaltrials.gov/ct2/show/NCT02562963, https://clinicaltrials.gov/ct2/show/NCT03198923, and https://clinicaltrials.gov/ct2/show/NCT01801852.
